# Health care costs of cardiovascular disease in China: a machine learning-based cross-sectional study

**DOI:** 10.3389/fpubh.2023.1301276

**Published:** 2023-11-06

**Authors:** Mengjie Lu, Hong Gao, Chenshu Shi, Yuyin Xiao, Xiyang Li, Lihua Li, Yan Li, Guohong Li

**Affiliations:** ^1^School of Public Health, Shanghai Jiao Tong University School of Medicine, Shanghai, China; ^2^Center for HTA, China Hospital Development Institute, Shanghai Jiao Tong University, Shanghai, China; ^3^Shanghai Municipal Health Commission, Shanghai, China; ^4^Department of Population Health Science and Policy, Icahn School of Medicine at Mount Sinai, New York, NY, United States; ^5^Icahn School of Medicine at Mount Sinai, Institute for Healthcare Delivery Science, New York, NY, United States; ^6^China Hospital Development Institute, Shanghai Jiao Tong University, Shanghai, China

**Keywords:** health care costs, cardiovascular disease, quantile regression forest, machine learning, financial burden

## Abstract

**Background:**

Cardiovascular disease (CVD) causes substantial financial burden to patients with the condition, their households, and the healthcare system in China. Health care costs for treating patients with CVD vary significantly, but little is known about the factors associated with the cost variation. This study aims to identify and rank key determinants of health care costs in patients with CVD in China and to assess their effects on health care costs.

**Methods:**

Data were from a survey of patients with CVD from 14 large tertiary grade-A general hospitals in S City, China, between 2018 and 2020. The survey included information on demographic characteristics, health conditions and comorbidities, medical service utilization, and health care costs. We used re-centered influence function regression to examine health care cost concentration, decomposing and estimating the effects of relevant factors on the distribution of costs. We also applied quantile regression forests—a machine learning approach—to identify the key factors for predicting the 10th (low), 50th (median), and 90th (high) quantiles of health care costs associated with CVD treatment.

**Results:**

Our sample included 28,213 patients with CVD. The 10th, 50th and 90th quantiles of health care cost for patients with CVD were 6,103 CNY, 18,105 CNY, and 98,637 CNY, respectively. Patients with high health care costs were more likely to be older, male, and have a longer length of hospital stay, more comorbidities, more complex medical procedures, and emergency admissions. Higher health care costs were also associated with specific CVD types such as cardiomyopathy, heart failure, and stroke.

**Conclusion:**

Machine learning methods are useful tools to identify determinants of health care costs for patients with CVD in China. Findings may help improve policymaking to alleviate the financial burden of CVD, particularly among patients with high health care costs.

## Introduction

Cardiovascular disease (CVD) refers to a group of disorders of the heart and blood vessels, including coronary heart disease (CHD), cerebrovascular disease, peripheral arterial disease, rheumatic heart disease, congenital heart disease, deep vein thrombosis, and pulmonary embolism. CVD is the leading cause of death globally (1). The World Health Organization (WHO) has reported that an estimated 17.9 million people died from CVD in 2019, representing 32% of all global deaths (2). Over three quarters of CVD deaths take place in developing countries (2). In China, it was estimated that about 330 million patients suffer from CVD and two out of every five deaths were due to CVD (3).

Besides the burden of morbidity and mortality, CVD results in substantial financial burden to patients and their families in China (4, 5). There is emerging evidence that CVD and other noncommunicable diseases can lead to poverty due to the high health care cost of treating the disease and high out-of-pocket expenditure among those who are uninsured or underinsured (6). Previous studies have assessed the health care cost of treating one type of CVD such as hypertension (7–10) and CHD (11–13) and mostly focused on developed countries such as the United States (6, 14–16). No studies, to the best of our knowledge, have assessed the health care costs of treating all types of CVD in China. The current study aims to fill this research gap by assessing the health care costs in patients with all types of CVD in China.

In previous health care cost analyses of CVD, mean-based models (e.g., generalized linear models with certain link functions for specific distributions) are often used to identify factors associated with the mean health care costs of CVD (17–19). These models evaluate the relationship between covariates and the mean outcome, assuming a uniform relationship across different percentiles of the cost distribution (20). However, this assumption does not always hold because the determinants of high health care costs may be different from those of low costs, and the effects of the determinants may vary across different parts of the cost distribution. The current study, instead, uses a machine learning approach to identify key determinants of health care costs in patients with CVD in China. We hypothesize that the determinants of health care cost vary from patients with high costs to those with low costs. The results would help healthcare professionals and policymakers design targeted interventions that may alleviate the financial burden of patients with CVD, their households, and the health care system.

## Methods

### Data sources and study population

This study used a cross-sectional cohort design. We exacted data from a survey of patients with CVD from 14 tertiary grade-A general hospitals in S City between 2018 and 2020. The list of hospitals is described in Supplementary Table S1. The survey collected patient information on their demography (e.g., age, sex), health conditions and comorbidities, medical service utilization, and health care costs.

Our sample included 28,213 adult patients ≥18 years. Excluding 591 patients with missing values on key variables, the final sample included 27,622 patients (Supplementary Figure S1). The International Classification of Diseases, 10th revision (ICD-10) codes I00-I99 and Q20-Q28 were used to identify patients with CVD (21). Specific types of CVD included congenital heart disease (ICD-10 codes Q20-Q28), coronary artery disease (CAD) (ICD-10 codes I25), heart failure (ICD-10 codes I50), myocardial infarction (MI) (ICD-10 codes I21), cardiomyopathy (ICD-10 codes I42-I43), hypertensive disease (ICD-10 codes I10-I15), and stroke (ICD-10 codes I60, I61, I63, I64) (Supplementary Table S2) (22). The survey data are de-identified. An exemption from ethical review has been approved by the Institutional Review Boards of School of Public Health at Shanghai Jiao Tong University School of Medicine.

### Outcomes

The outcomes of interest included total health care cost, which consists of insurance-covered cost and out-of-pocket cost. We also considered health care costs by treatment category, including the cost of comprehensive medical service, diagnosis, treatment, medication, and medical consumables.

### Covariates

The covariates included age, sex, marital status, residency (living in S City, living outside of S City), employment status (retired, employed, unemployed), common diagnosis (CHD, CAD, heart failure, MI, cardiomyopathy, hypertensive disease and stroke), cardiogenic comorbidity (yes, no), non-cardiac comorbidity (yes, no), number of comorbidities (0, 1, 2, 3, 4, ≥5), number of medical procedures, level of operation (I, II, III, IV), length of hospital stay, payment method (Urban Employee Basic Medical Insurance (UEBMI), New Rural Cooperative Medical Scheme/Urban Resident Basic Medical Insurance (NRCMS/URBMI), full self-payment and others), admission type (emergency and outpatient), and proportion of out-of-pocket.

### Statistical analysis

To describe the distribution of health care costs, we first calculated the means of different quantiles of annual health care costs by demographic characteristics and compared the differences. We also calculated the Gini coefficient, a measure of inequality, for health care costs, the proportion of high health care costs (top 10%) in total costs. A high Gini coefficient suggests inequality in health care costs. We examined the statistical differences between high- (top 10%) and low-cost patients (bottom 10%) by between-group difference tests. We used recentered influence functions (RIFs) to estimate the small changes in the distribution of independent variables on the distributional measure of interest such as Gini coefficient (23).

We used quantile regression forests (QRFs) to identify determinants of health care costs across patients with different quantiles of CVD costs. Quantile regression (QR) can estimate how specific quantiles or percentiles of the distribution of the outcome variable vary with covariates. It is robust against outliers and is more informative for a skewed distribution than mean-based regression (24). QRFs is an extension of QR and has been applied in health care research for its prediction accuracy (25). QRFs utilizes the infrastructure of random forests and is a nonparametric model for conditional quantile estimation.

Specifically, we implemented a backward stepwise variable selection algorithm developed by Hu et al. (26), based on the variable importance scores generated by QRFs to determine the key factors for the 10th, 50th, and 90th quantiles, respectively, representing the low-, and high cost threshold of health care costs in patients with CVD (25). We computed the importance score of a QRFs for each covariate based on the “mean decrease in accuracy” (20). In a QRFs ensemble, each tree has an out-of-bag (OOB) sample that was left out from tree construction for assessing the predictive performance of the tree model (25). An iterative process was carried out for variable selection. Each time, we removed the least important variable and rebuilt a QRFs model with the remaining variables and recorded the out-of-bag average quantile loss (AQL) until no variable was left. AQL was used for the evaluation of model performance. Finally, we performed a weighted quantile regression (QR) to quantify the effects of each selected factor on different quantiles of health care costs in patients with CVD. Taking into account the variance across the specific CVD diseases, we performed subgroup analyses of CAD and stroke, the two diseases with the highest prevalence. As a sensitivity analysis, we also used GLM to estimate the effect of the key factors on the medical expenditures by the mean-based approach.

All statistical analyses were performed by R version 4.2.2. QRFs models were built using the “quantregForest” R package. A *p* value from two-sided test <0.05 was considered statistically significant.

## Results

### Population characteristics and distribution of health care costs

Out of 27,622 patients with CVD, 10,614 (38%) were female and 19,427 (70%) lived in S City. The mean age of the patients was 64.14 (14.09) years. The proportion of males among the top 10% of patients was higher than among the bottom 10% of patients (64% vs. 56%), and the same applied to the proportion of non-native (46% vs. 31%). Other population characteristics were summarized in Table 1.

**Table 1:** Demographic, disease characteristics and medical service utilization of inpatients with CVDs (*n* = 27,622).

Characteristics	All inpatients	The bottom 10%	The bottom 10–50%	The top 10–50%	The top 10%
**Demography characteristics**					
**Sex, *n* (%)**					
Male	17,008 (62)	1,537 (56)	6,441 (58)	7,265 (66)	1,765 (64)
Female	10,614 (38)	1,225 (44)	4,608 (42)	3,784 (34)	997 (36)
Age, mean (SD)	64.14 (14.09)	63.04 (15.73)	64.50 (13.94)	64.25 (14.19)	63.40 (12.38)
**Marriage, *n* (%)**					
Unmarried	1,168 (4)	132 (5)	312 (5)	473 (4)	84 (3)
Married	23,369 (85)	2,425 (88)	2,425 (88)	9,298 (84)	2,337 (85)
Widowed or divorced	3,085 (11)	205 (7)	205 (7)	1,278 (12)	341 (12)
**Residence, *n* (%)**					
Non-native	8,195 (30)	845 (31)	2,565 (23)	3,506 (32)	1,279 (46)
Native	19,427 (70)	1,917 (69)	8,484 (77)	7,543 (68)	1,483 (54)
**Occupation, *n* (%)**					
Retired employed	10,385 (38)	975 (35)	4,460 (40)	4,064 (37)	886 (32)
Employed	16,100 (58)	1,688 (61)	6,201 (56)	6,471 (59)	1,740 (63)
Unemployed	1,137 (4)	99 (4)	388 (4)	514 (5)	136 (5)
**Disease characteristics**					
**Common disease, *n* (%)**					
Hypertensive disease	1,521 (6)	336 (12)	951 (9)	216 (2)	18 (1)
Coronary artery disease (CAD)	7,239 (26)	592 (21)	3,307 (30)	2,964 (27)	376 (4)
Cardiomyopathy	291 (1)	60 (2)	133 (1)	53 (0)	45 (2)
Heart failure	542 (2)	55 (2)	281 (4)	170 (2)	36 (1)
Stroke	3,177 (12)	100 (4)	1,588 (15)	1,068 (10)	321 (12)
Congenital heart disease (CHD)	771 (3)	79 (3)	224 (2)	383 (3)	85 (3)
Myocardial infarction (MI)	946 (3)	15 (1)	92 (1)	761 (7)	78 (3)
Comorbidity type					
**Cardiogenic comorbidity, *n* (%)**					
No	12,167 (44)	1,415 (51)	5,304 (48)	4,519 (41)	929 (34)
Yes	15,455 (56)	1,347 (49)	5,745 (52)	6,530 (59)	1,833 (66)
**Non-cardiac comorbidity, *n* (%)**					
No	19,313 (70)	1,884 (68)	7,335 (66)	7,906 (72)	2,188 (79)
Yes	8,309 (30)	878 (32)	3,714 (34)	3,143 (28)	574 (21)
**No. of comorbidities, *n* (%)**					
0	3,852 (14)	537 (19)	1,588 (14)	1,373 (12)	354 (13)
1	4,352 (16)	487 (18)	1,716 (16)	1,703 (15)	446 (16)
2	5,079 (18)	520 (19)	1,987 (18)	2,060 (19)	512 (19)
3	4,448 (16)	424 (15)	1,715 (16)	1,860 (17)	446 (16)
≥4	9,894 (36)	794 (29)	4,043 (37)	4,053 (37)	1,004 (36)
**No. of medical procedures**					
0	7,920 (29)	2,270 (82)	4,380 (40)	1,247 (11)	23 (1)
1	8,493 (31)	424 (15)	4,926 (45)	2,777 (25)	366 (13)
2	4,455 (16)	57 (2)	1,089 (10)	2,757 (25)	552 (20)
3	3,185 (12)	9 (0)	571 (5)	1,997 (18)	608 (22)
≥4	3,569 (13)	2 (0)	83 (1)	2,271 (21)	1,213 (44)
**Level of medical procedure, *n* (%)**					
I	3,119 (15)	517 (60)	2,157 (31)	421 (4)	24 (1)
II	4,167 (21)	123 (14)	2,223 (32)	1,644 (17)	177 (7)
III	6,003 (30)	123 (14)	2,381 (34)	2,943 (31)	556 (21)
IV	6,893 (34)	103 (12)	285 (4)	4,586 (48)	1,919 (72)
**Medical service utilization**					
Length of hospital stay, mean (SD)	7.73 (9.41)	3.69 (2.43)	5.81 (4.06)	8.32 (8.24)	17.09 (20.54)
**Medical payment, *n* (%)**					
UEBMI	16,071 (58)	1,551 (56)	6,983 (63)	6,284 (57)	1,253 (45)
NRCMS/URBMI	2,595 (9)	258 (9)	1,062 (10)	977 (9)	298 (11)
Full out-of-pocket	6,473 (23)	761 (28)	2,357 (21)	2,579 (23)	776 (28)
Others	2,483 (9)	192 (7)	647 (6)	1,209 (11)	435 (16)
**Admission type, *n* (%)**					
Emergency	6,649 (24)	365 (13)	2,381 (22)	3,012 (27)	891 (32)
Outpatient	20,973 (76)	2,397 (87)	8,668 (78)	8,037 (73)	1,871 (68)
Proportion of out-of-pocket, *n* (%)	33.06 (37.29)	30.71 (40.92)	29.16 (37.61)	35.27 (35.53)	42.22 (36.81)

Level of medical procedure: I: a variety of surgeries with low technical difficulty, simple surgical procedure and low risk; II: all kinds of surgery with average technical difficulty, uncomplicated surgical procedure and medium risk. III: all kinds of surgery with relatively high technical difficulty, complicated surgical process and high risk. IV: all kinds of surgery with high technical difficulty, complicated surgical process and high risk. The level of operation is the highest of all operations. UEBMI, urban employee basic medical insurance; NRCMS, new rural cooperative medical system; URBMI, urban resident basic medical insurance.

**Figure 1:**
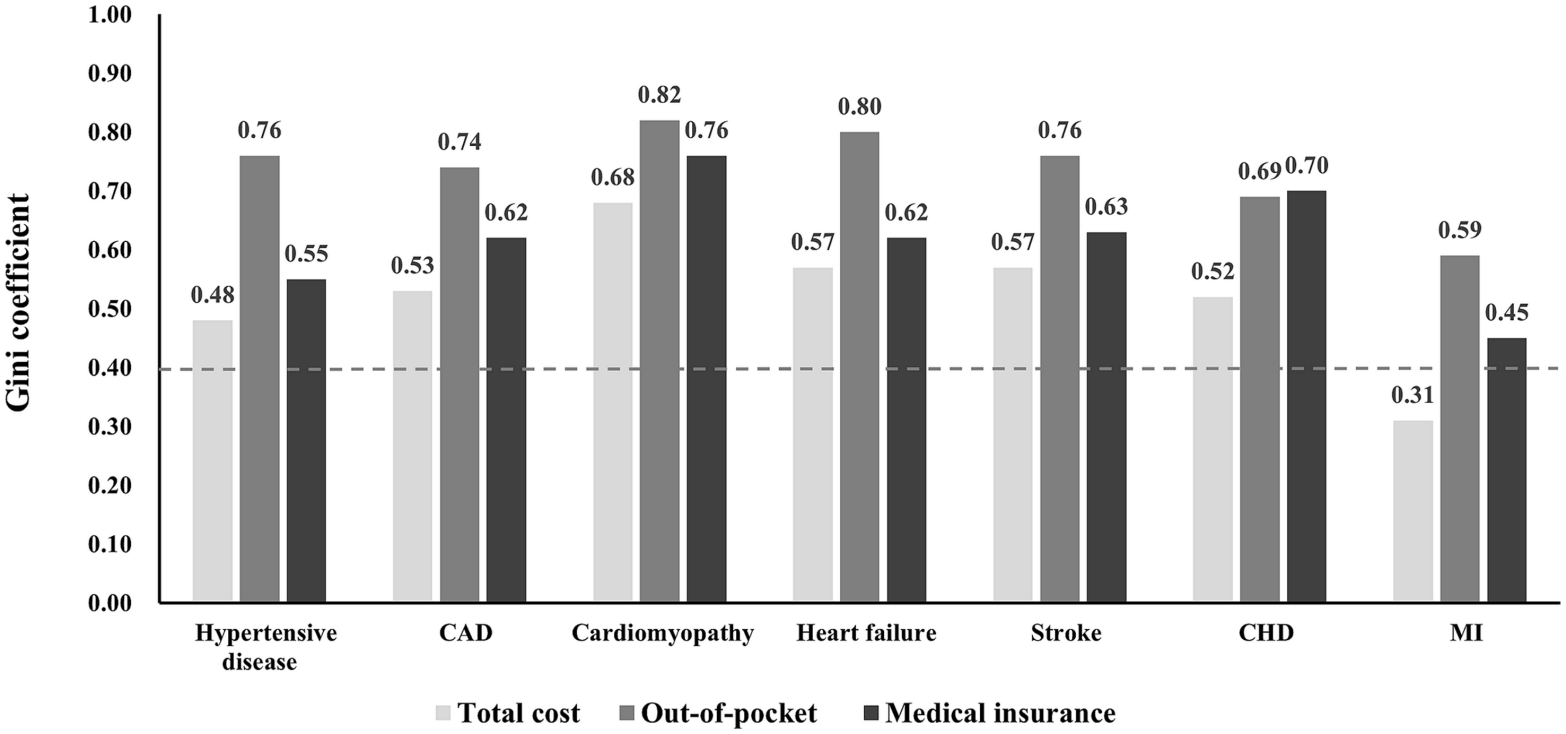
Gini coefficient of health care cost.

Nearly 38% of the health care costs for treating CVD were generated by the top 10% of patients and 1% by the bottom 10%. The Gini coefficient of health care costs in patients with CVD was 0.56, that of out-of-pocket cost was 0.74, and that of medical insurance cost was 0.65, indicating a very high concentration. The Gini coefficient of health care costs for additional factors were showed in Supplementary Table S3. Among all types of CVD, the Gini coefficients of the costs exceeded 0.4 except for MI. The Gini coefficient of cardiomyopathy was the largest, reaching 0.68 in total cost, 0.82 in out-of-pocket, and 0.76 in medical insurance, which was much higher than the average (Figure 1).

The average health care cost was 41,282 CNY, while the 10th, 50th, and 90th of the costs were 6,103 CNY, 18,105 CNY, and 98,637 CNY, respectively. The average health care costs for each type of CVD were presented in Supplementary Table S4. Among them, myocardial infarction (MI) had the highest average annual per-person cost (56,118 CNY). Hypertensive disease had the highest cost (4,533 CNY) in the low-cost group, while cardiomyopathy was highest in the high-cost group. Coronary artery disease (CAD) accounted for the highest proportion (21.04%) of total costs (Supplementary Table S4). The bottom 10% of inpatients had a higher proportion of insurance coverage, accounting for 93.8%, while the top 10% had 72.3%. Among the specific categories of health care costs, the bottom 10% of patients had the highest proportion of diagnostic costs, reaching 62.8%, while the top 10% of patients had the highest proportion of medical consumables, reaching 62.9% (Supplementary Table S5).

### Variable selection and rank of importance in different quantiles

**Figure 2:**
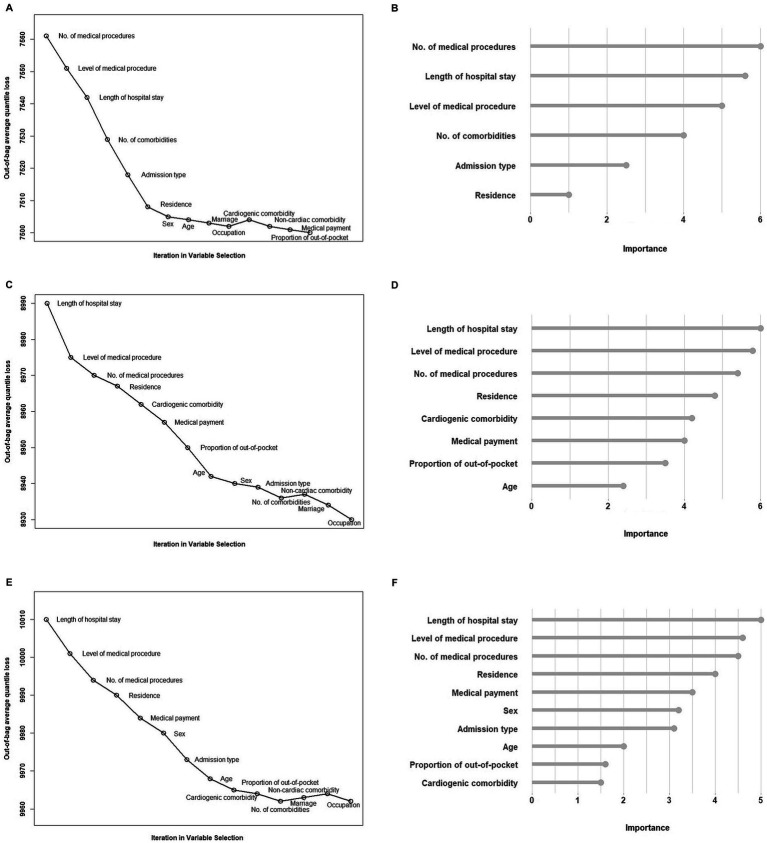
**(A)**, **(C)** and **(E)** plotted the estimated out-of-bag AOL from every QRFs model built at each iteration in the backward stepwise algorithm for the 10th, 50th and 90th quantiles of health care costs in patients with CVD. **(B)**, **(D)** and **(F)** plotted the importance scores for the selected key factors for the 10th, 50th and 90th quantiles of health care costs in patients with CVD.

Figures 2A,C,E showed the estimated out-of-bag AOL from every QRFs model built at each iteration in the backward stepwise algorithm for the 10th, 50th, and 90th quantiles of health care costs in patients with CVD. Number of operations, level of operation, inpatient length of stay, admission type and residence were selected as important factors for patients with both the 10th percentile, 50th percentile, and 90th percentile healthcare costs. Number of comorbidities was selected as an important factor only for patients with the 10th percentile cost, while sex, age, medical payment and proportion of self-payment were selected only for patients with the 90th percentiles costs. Figures 2B,D,F plotted the importance scores for the selected key factors for the 10th, 50th, and 90th quantiles of health care costs in patients with CVD. The variable selection of the identified key factors and the estimated effect of selected key factors for the 10th, 50th, and 90th percentile of health care costs with CAD and stroke were presented in Supplementary Figures S2, S3 and Supplementary Tables S7, S8.

### Estimated effect of selected key factors

Table 2 presented the estimated effects of the identified key determinants on total health care costs in patients with CVD. Compared to females, males were associated with higher costs among the high-cost groups (1,615 CNY, 95%CI: 348 CNY, 2,883 CNY). The cost would increase by 162 CNY (95% CI: 117 CNY, 207 CNY) per year of age among the high-cost group, but age was not significantly associated with health care costs in the low-cost group. The number of comorbidities was significantly associated with health care costs among the low-cost group. Patients with one, two, three, four or more comorbidities would have additional costs of 2,026 CNY (95% CI: 1,582 CNY, 2,471 CNY), 2,232 CNY (95% CI: 1,842 CNY, 2,622 CNY), 2,564 CNY (95% CI: 2,171 CNY, 2,957 CNY), and 2,635 CNY (95% CI: 2,267 CNY, 3,002 CNY), respectively, compared to those with no comorbidities. However, the number of comorbidities was not associated with costs among the high-cost groups. Payment methods were associated with costs in the high-cost groups, but not in the low-cost groups. Compared to UEBMI, NRCMS/URBMI was associated with higher costs (5,545 CNY, 95% CI: 660 CNY, 10,430 CNY), while this association was opposite in fully self-payment (−5,673 CNY, 95% CI: −7,841 CNY, −3,505 CNY). For every 1% increase in the proportion of self-payment, the costs increased by 134 CNY (95% CI: 100 CNY, 167 CNY) among the high-cost groups. Results from the sensitivity analysis using mean-based approach (GLM) were presented in Supplementary Table S9.

**Table 2:** Estimated effect of selected key factors on the medical expenditures using quantile regressions.

Characteristics	10th Quantile	50th Quantile	90th Quantile
**Demography characteristics**			
**Sex**			
Female vs. male	Not select	Not select	−1744.0 (−2976.8, −511.2)
Age	Not select	32.9 (19.1, 46.7)	138.7 (94.2, 183.2)
**Marriage: ref. = Unmarried**			
Married	Not select	Not select	Not select
Widowed or divorced	Not select	Not select	Not select
**Residence**			
Native vs. non-native	−487.5 (−722.5, −252.5)	−2660.8 (−3389.4, −1932.1)	−46629.7 (−6753.8, −2505.6)
**Occupation: ref. = Retired**			
Employed	Not select	Not select	Not select
Unemployed	Not select	Not select	Not select
**Cardiogenic comorbidity**			
Yes vs. No	Not select	1331.2 (765.5, 1897.0)	2955.8 (1507.5, 4404.1)
**Non-cardiac comorbidity**			
Yes vs. No	Not select	Not select	Not select
**No. of comorbidities: ref. = 0**			
1	2026.4 (1581.6, 2471.2)	Not select	Not select
2	2231.9 (1842.3, 2621.5)	Not select	Not select
3	2563.9 (2171.3, 2956.5)	Not select	Not select
≥4	2634.7 (2267.3, 3002.1)	Not select	Not select
**No. of medical procedures: ref. = 0**			
1	3556.0 (1899.7, 5212.2)	4118.0 (3604.4, 4631.5)	4301.3 (3249.6, 5352.9)
2	6793.5 (5127.7, 8459.4)	15314.7 (13727.0, 16902.4)	25434.5 (22903.8, 27965.2)
3	9264.9 (7590.0, 10939.8)	20719.0 (18832.8, 22605.2)	21383.0 (17899.2, 24866.8)
≥4	21279.9 (19660.8, 22899.0)	36196.5 (34394.6, 37998.5)	43420.3 (38433.7, 48406.8)
**Level of medical procedure: ref. = I**			
II	2605.9 (2320.3, 2891.5)	1228.1 (832.1, 1624.0)	31129.8 (28348.9, 33910.7)
III	2717.5 (2430.0, 3005.0)	6127.4 (5381.0, 6873.7)	39079.9 (36676.1, 41483.6)
IV	15668.0 (14480.0, 16855.9)	30406.2 (29288.0, 31524.3)	73859.6 (70215.4, 77503.8)
**Medical service utilization**			
Length of hospital stay	8288.0 (7516.5, 9059.5)	27299.2 (26155.8, 28442.6)	446134.6 (43871.2, 48398.0)
**Medical payment: ref. = UEBMI**			
NRCMS/URBMI	Not select	−36.8 (−739.1, 665.5)	5459.9 (1296.5, 9623.3)
Full out-of-pocket	Not select	−3333.9 (−3976.2, −2691.5)	−5721.2 (−7699.5, −3742.8)
Others	Not select	1324.3 (13.3, 2635.3)	918.7 (−1923.4, 3760.8)
**Admission type**			
Outpatient vs. Emergency	−907.6 (−1243.7, −571.5)	Not select	−41128.6 (−5878.9, −2378.2)
Proportion of out-of-pocket	Not select	40.8 (32.6, 48.9)	1377.3 (106.4, 168.3)

## Discussion

The determinants of health care costs in patients with CVD in China are poorly known. Using data from a survey of patients with CVD from 14 large hospitals in China, we identified the determinants of health care costs in patients CVD and assessed their effects on the costs. The results showed that the health care costs in urban patients with CVD in China were highly concentrated in small groups of patients, similar to the empirical evidence (4, 27–29). The top 10% of inpatients accounted for approximately 38% of annual health care costs, and only 8% of the insured (UEBMI/NRCMS/URBMI) consumed close to 61% of annual inpatient medical insurance expenditures, which was generally consistent with the conclusions of some studies about other diseases in China (4, 30). Our study found that there was no difference in the concentration of total costs among age groups, but the concentration of out-of-pocket expenses and medical insurance expenses was completely opposite. The older adults have the highest concentration degree of out-of-pocket expenses and the lowest concentration degree of medical insurance expenses, although some evidence suggesting that concentration decreased with age (4, 31). Sex differences existed in the concentration of inpatient medical expenditures, with females having a greater concentration than males at all expense categories. Among the seven common diseases, patients with cardiomyopathy had the highest concentration and ones with MI had the lowest. Our results showed that high-cost inpatients were male and older, which explained the increase in the concentration of inpatient health care costs with population aging.

Employing the rigorous methodology to identify determinants for health care costs is important to informing the debate regarding how to improve health care value overall and address variation in health care costs in patients with CVD among low-cost and high-cost groups. It is particularly critical given the highly skewed distribution of health care costs with CVD in China. In previous studies, key factors of health care costs with CVD were often found using mean-based regression or for one of common diseases (9, 15, 19). To address the limitations, we used a machine learning with a principled backward stepwise algorithm (26) to identify determinants for varied levels of health care costs with CVD through a large survey with many covariates. We found that most of the key factors were the same across the quantiles, however, number of comorbidities was selected only the 10th percentile, while sex, age, medical payment and proportion of self-payment were selected only the 90th percentiles. Through selecting the key factors based on various quantiles of health care costs and ranking the relative importance, our study showcased the a more appropriate method for a detailed understanding of how determinants explain the variability in different parts of the health care cost distribution.

One major contribution of our study was to quantify the drivers for health care costs in patient with CVD in different cost groups. Previous studies have confirmed the basic consensus that some factors that elevated the probability of being a high-cost users (28, 30, 32, 33). However, with only qualitative assumptions, the impact of potential drivers on changes in concentration remains ambiguous, and a rigorous quantitative assessment lacking. To bridge this gap, we examined the drivers of health care costs in patient with CVD using the quantile regression forests. Our results showed that if the proportion of inpatients with cardiomyopathy increased by 10%, the predicted Gini coefficient would increase by 2.11%, and the costs gap would increase 2.08%. This suggested that the low prevalence of serious diseases, represented by cardiomyopathy, but accompanied by high-cost characteristics, was an important driver of health care cost concentration. In recent years, the burden of disease in China has shifted considerably, with the epidemiological transition from acute diseases, to chronic disease, such as cardiovascular diseases (34). Furthermore, the prevalence of chronic diseases, and trends of specific chronic diseases, has increased (35). For example, cardiovascular hospitalization costs increased by more than 20% annually since 2004 (1), stroke prevalence increased by 155% and the incidence increased by 31.6% in rural areas from 1980s to 2013 (36). The prevalence and spending on cardiovascular diseases will continue to rise as China’s demographics reflect population aging, prolonged life expectancy, increased expectation of medical care, and declining mortality rates, as well as the accumulation of risk factors.

We also found that these determinants did not uniformly impact the health care costs with linking the selected key determinants to health care costs using a weighted quantile regression. For example, number of comorbidities was selected only the 10th percentile. This finding might be surprising, given that it had been well documented that the high burden of comorbidities (37, 38). However, the magnitude of the relative difference was most profound at the lower percentile. It might be that the high cost of health care for those with CVD is more prominent among individuals with lower health care costs and has less of a differential impact on costs among those with higher costs and more complex conditions and care needs. In addition, medical payment method was selected only the 90th percentile. Compared with UEBMI, hospitalization costs were higher using the NRCMS/URBMI and lower with the full self-payment approach. This disproportionality in the effect estimates was often ignored in frequently used mean-based methods, potentially leading to biased conclusions.

Results from our study might also provide important insights for the development of tailored interventions to reduce potentially inappropriately high health care costs of CVD while maintaining or improving the care quality. For example, residence was significantly associated with higher percentile of health care costs of CVD. Due to the rich medial resources and top-notch medical technology, patients who seek medical treatment outside the city often suffer from more complex or severe diseases. Among the patients hospitalized in other provinces, 9.8% had Level-I medical procedure and 17.5% had Level-II medical procedure. These patients may be able to receive medical treatment locally. In addition, patients hospitalized in other provinces also incur more indirect costs, such as accommodation and transportation. Developing strategies by policymakers to reduce unnecessary or undesired treatments and related spending for out-of-town patients is warranted.

There are several limitations in the study. First, we conducted the large survey in almost all “AAA” general hospitals in S City, China. The findings generalized to the cities with rich medical resources but not the whole country. Second, we were not able to build causality in the relationships between health care costs and demographics, disease characteristics, and medical service utilization due to the cross-sectional nature of the survey data (39). However, our study identified determinants important for different quantiles of health care costs with CVD and can serve as a groundwork for future causal inference research in cost analysis. Third, we cannot evaluate other important variables that were not included in the study, either not measured or not collected in the survey, such as treatment quality or prognosis due to the lack of the follow-up. Despite the potential omitted variables, by using machine learning approach on a large sample that included individuals across demographics, residential information, comorbidity, surgery or operation information, insurance types, we believed our study deepens the understanding of the complex web of drivers and expands current research on CVD health care costs.

## Conclusion

This study assessed the health care costs of treating all types of CVD in China and identified key determinants of high health care costs. To our knowledge, this is the first study assessing the economic burden of all types of CVD in China. Patients with high health care costs were more likely to be older, male, and have a longer length of hospital stay, more comorbidities, more complex medical procedures, and emergency admissions. Higher health care costs were also associated with specific CVD types such as cardiomyopathy, heart failure, and stroke. All of these findings may provide important insights for the development of tailored interventions to alleviate the financial burden of CVD in China, particularly among patients with high health care costs.

## Data availability statement

The raw data supporting the conclusions of this article will be made available by the authors, without undue reservation.

## Ethics statement

The studies involving humans were approved by the institutional review board of School of Public Health at Shanghai Jiao Tong University School of Medicine on February 20, 2020 (IRB# SJUPN-202008). The studies were conducted in accordance with the local legislation and institutional requirements. The participants provided their written informed consent to participate in this study.

## Author contributions

ML: Methodology, Validation, Writing – original draft. HG: Data curation, Resources, Writing – review & editing. CS: Writing – original draft. YX: Supervision, Writing – original draft. XL: Data curation, Writing – review & editing. LL: Writing – review & editing. YL: Conceptualization, Methodology, Writing – review & editing. GL: Funding acquisition, Project administration, Writing – review & editing.
