# Supplementary material for: Health care costs of cardiovascular disease in China: a machine learning-based cross-sectional study
Source: Front Public Health. 2023 Nov 6;11:1301276. doi: 10.3389/fpubh.2023.1301276 (PMC10657803; doi:10.3389/fpubh.2023.1301276)
Supplement: Supplementary file 1 [file Table_1.docx]

**Supplemental Document**

**Using Machine Learning to Identify Determinants of Health Care Costs in Patients with Cardiovascular Disease in China**


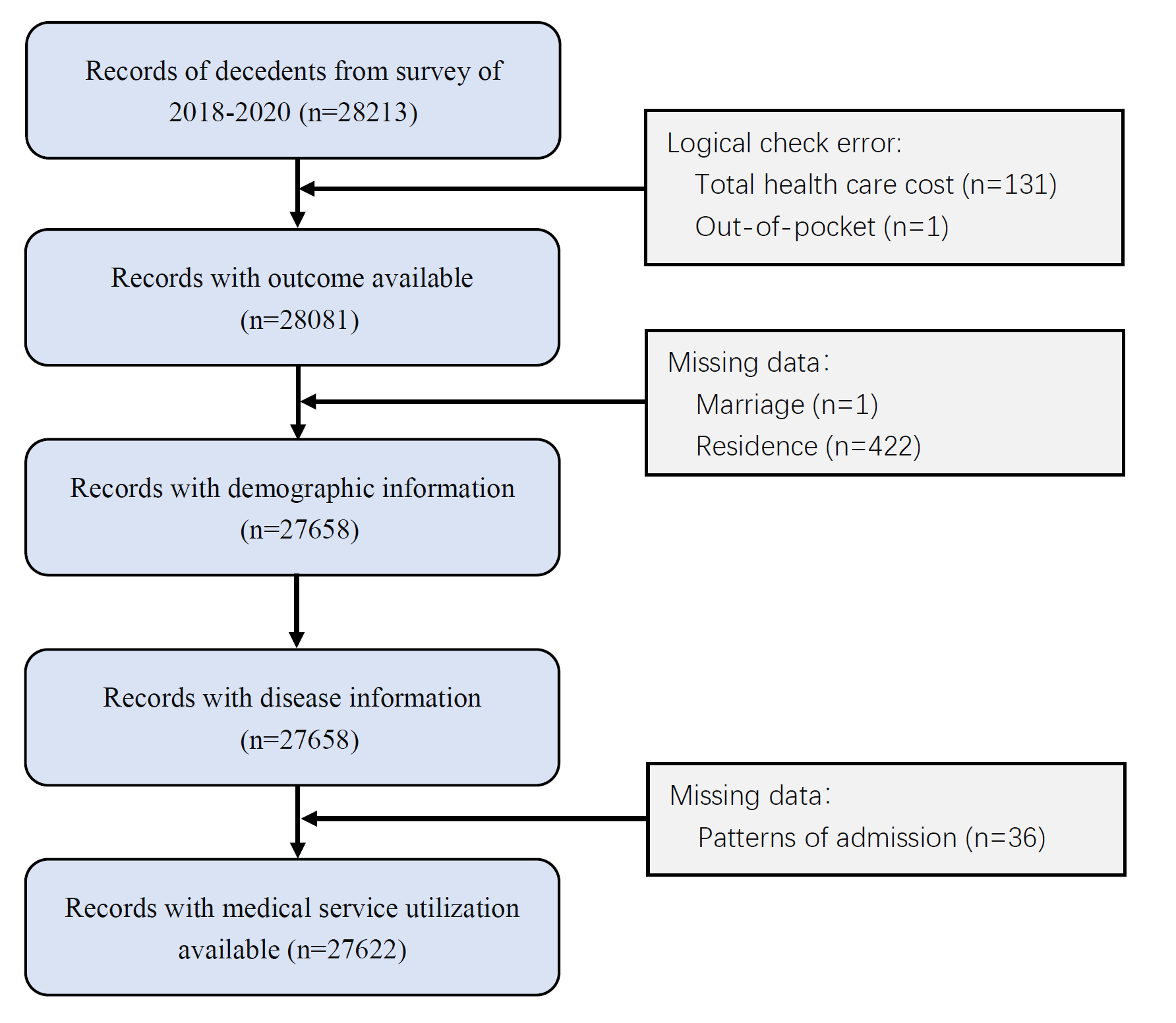


Figure S1. Flow chart of study sample


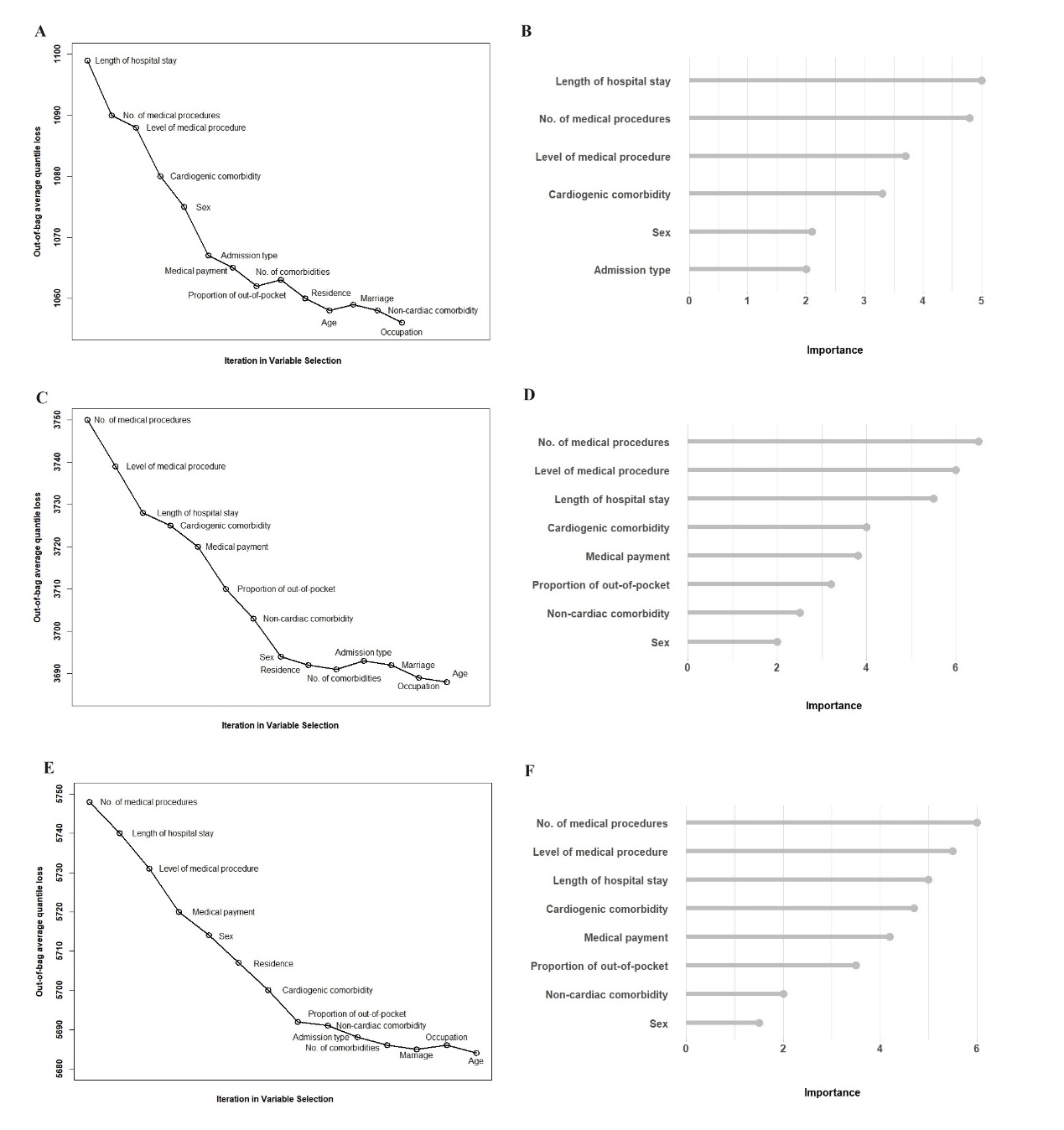


Figure S2. The variable selection of the identified key factors for the 10^th^, 50^th^ and 90^th^ percentile of health care costs with CAD


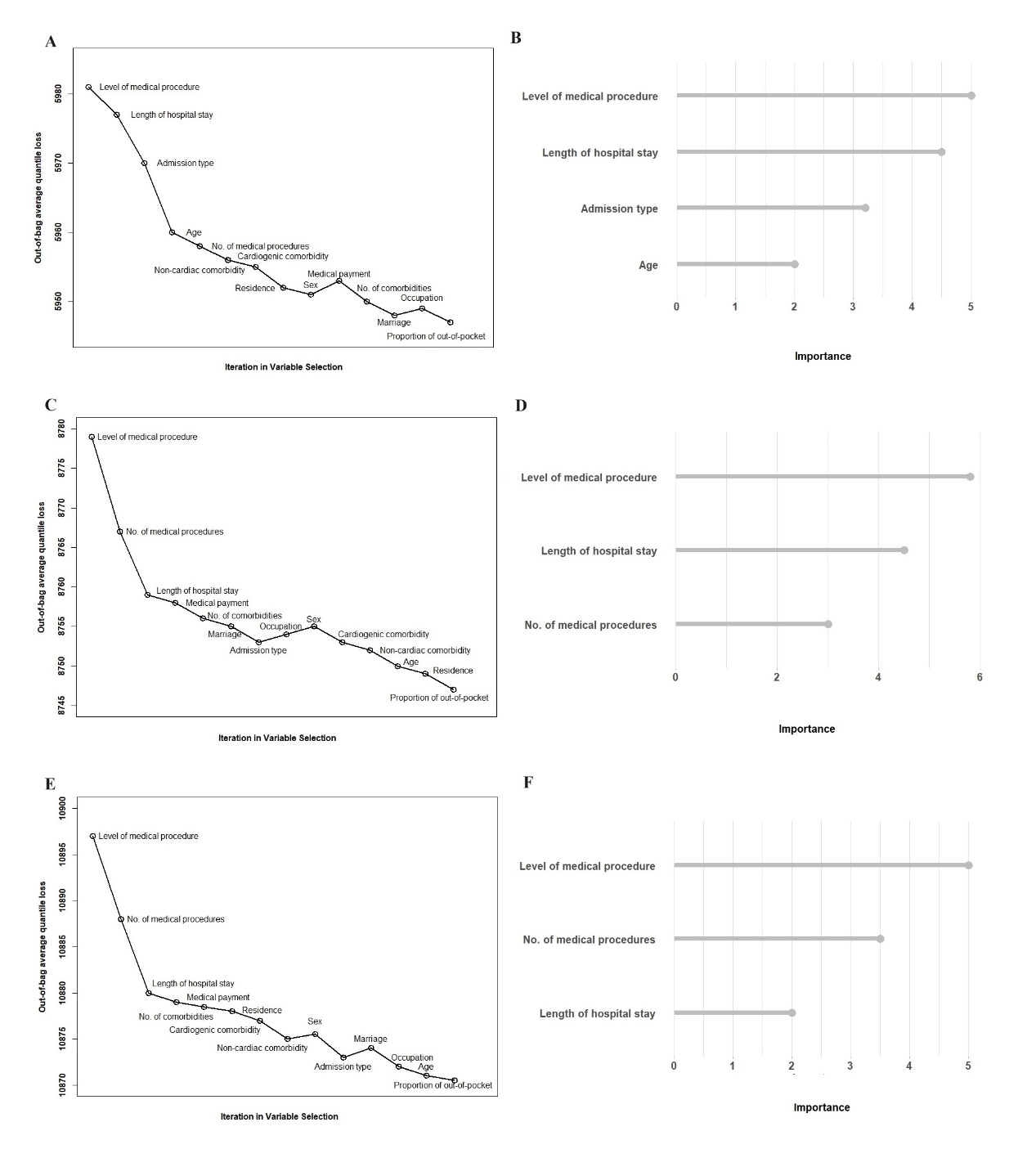


Figure S3. The variable selection of the identified key factors for the 10th and 90th percentile of health care costs with stroke

Table S1. Summary of surveyed hospitals and population

Note: About 10% of the total CVDs in each hospital were surveyed.

| No. | Hospital | N (%) |
| --- | --- | --- |
| 1 | Huashan Hospital Affiliated to Fudan University | 1284 (5.1) |
| 2 | Zhongshan Hospital Fudan University | 6136 (24.3) |
| 3 | Shanghai Changzheng Hospital | 665(2.7) |
| 4 | Shanghai Changhai Hospital | 953 (3.8) |
| 5 | Huadong Hospital Affiliated to Fudan University | 904 (3.6) |
| 6 | Ninth People’s Hospital Affiliated to Shanghai Jiao Tong University School of Medicine | 1100 (4.4) |
| 7 | Renji Hospital Affiliated to Shanghai Jiao Tong University School of Medicine | 1073 (4.3) |
| 8 | Ruijin Hospital Affiliated to Shanghai Jiao Tong University School of Medicine | 2248 (8.9) |
| 9 | Xinhua Hospital Affiliated to Shanghai Jiao Tong University School of Medicine | 1717 (6.8) |
| 10 | Sixth People’s Hospital Affiliated to Shanghai Jiao Tong University School of Medicine | 1553 (6.2) |
| 11 | Tenth People’s Hospital of Tongji University | 1225 (4.9) |
| 12 | Shanghai General Hospital Affiliated to Shanghai Jiao Tong University School of Medicine | 2083 (8.3) |
| 13 | Shanghai Tongji Hospital Affiliated to Tongji University | 1880 (7.5) |
| 14 | Shanghai Dongfang Hospital Affiliated to Tongji University | 2395 (9.5) |

| Table S2. ICD-10 diagnosis codes | | |
| --- | --- | --- |
| Disease | Code | Block |
| Cardiovascular diseases (CVDs) | I00-I02 | Acute rheumatic fever |
|  | I05-I09 | Chronic rheumatic heart disease |
|  | I10-I15 | Hypertensive disease |
|  | I20-I25 | Ischemic heart disease |
|  | I26-I28 | Pulmonary heart disease and diseases of pulmonary circulation |
|  | I30-I52 | Other forms of heart disease |
|  | I60-I69 | Cerebrovascular disease |
|  | I70-I79 | Disease of arteries, arterioles and capillaries |
|  | I80-I89 | Diseases of veins, lymphatic vessels and lymph nodes, not elsewhere classified |
|  | I95-I99 | Other and unspecified disorders of the circulatory system |
|  | Q20-Q26 | Congenital malformations of the circulatory system |
| Hypertensive disease | I10 | Essential (primary) hypertension |
|  | I11 | Hypertensive heart disease |
|  | I12 | Hypertensive renal disease |
|  | I13 | Hypertensive heart and renal disease |
|  | I15 | Secondary hypertension |
| Coronary artery disease (CAD) | I25 | Chronic ischemic heart disease |
| Cardiomyopathy | I42 | Cardiomyopathy |
|  | I43 | Cardiomyopathy in diseases classified elsewhere |
| Heart failure | I50 | Heart failure |
| Stroke | I60 | Subarachnoid haemorrhage |
|  | I61 | Intracerebral haemorrhage |
|  | I63 | Cerebral infarction |
|  | I64 | Stroke, not specified as haemorrhage or infarction |
| Congenital heart disease (CHD) | Q20 | Congenital malformations of cardiac chambers and connections |
|  | Q21 | Congenital malformations of cardiac septa |
|  | Q22 | Congenital malformations of pulmonary and tricuspid valves |
|  | Q23 | Congenital malformations of cardiac septa |
|  | Q24 | Other congenital malformations of heart |
|  | Q25 | Congenital malformations of great arteries |
|  | Q26 | Congenital malformations of great veins |
|  | Q27 | Other congenital malformations of peripheral vascular system |
|  | Q28 | Other congenital malformations of circulatory system |
| Myocardial infarction (MI) | I21 | Acute myocardial infarction |

Table S3. Gini coefficient of health care cost

| Characteristics | Total cost | Out-of-pocket | Medical insurance |
| --- | --- | --- | --- |
| All patients | 0.56 | 0.74 | 0.65 |
| Demography characteristics |  |  |  |
| Sex |  |  |  |
| Male | 0.55 | 0.73 | 0.64 |
| Female | 0.58 | 0.76 | 0.66 |
| Age, years |  |  |  |
| 18-59 | 0.56 | 0.73 | 0.69 |
| 60-79 | 0.56 | 0.74 | 0.64 |
| ≥80 | 0.55 | 0.77 | 0.58 |
| Marriage |  |  |  |
| Unmarried | 0.56 | 0.71 | 0.68 |
| Married | 0.56 | 0.74 | 0.64 |
| Residence |  |  |  |
| Non-Shanghai | 0.54 | 0.69 | 0.76 |
| Shanghai | 0.56 | 0.73 | 0.59 |
| Occupation |  |  |  |
| Retired employed | 0.57 | 0.77 | 0.60 |
| Employed | 0.56 | 0.73 | 0.67 |
| Unemployed | 0.53 | 0.69 | 0.71 |
| Disease characteristics |  |  |  |
| Common disease |  |  |  |
| Hypertensive disease | 0.48 | 0.76 | 0.55 |
| CAD | 0.53 | 0.74 | 0.62 |
| Cardiomyopathy | 0.68 | 0.82 | 0.76 |
| Heart failure | 0.57 | 0.80 | 0.62 |
| Stroke | 0.57 | 0.76 | 0.63 |
| CHD | 0.52 | 0.69 | 0.70 |
| MI | 0.31 | 0.59 | 0.45 |
| Comorbidity type |  |  |  |
| Cardiogenic comorbidity |  |  |  |
| No | 0.58 | 0.74 | 0.67 |
| Yes | 0.55 | 0.75 | 0.63 |
| Non-cardiac comorbidity |  |  |  |
| No | 0.56 | 0.74 | 0.64 |
| Yes | 0.57 | 0.74 | 0.65 |
| No. of comorbidities |  |  |  |
| 0 | 0.58 | 0.72 | 0.70 |
| 1 | 0.57 | 0.75 | 0.67 |
| 2 | 0.55 | 0.74 | 0.64 |
| 3 | 0.54 | 0.74 | 0.62 |
| ≥4 | 0.56 | 0.74 | 0.63 |
| No. of operations |  |  |  |
| 0 | 0.42 | 0.73 | 0.53 |
| 1 | 0.56 | 0.76 | 0.63 |
| 2 | 0.44 | 0.65 | 0.54 |
| 3 | 0.40 | 0.62 | 0.56 |
| ≥4 | 0.35 | 0.60 | 0.53 |
| Level of operation |  |  |  |
| I | 0.41 | 0.77 | 0.49 |
| II | 0.53 | 0.74 | 0.62 |
| III | 0.50 | 0.69 | 0.61 |
| IV | 0.36 | 0.59 | 0.52 |
| Medical service utilization |  |  |  |
| Inpatient length of stay |  |  |  |
| 0-7 days | 0.55 | 0.74 | 0.63 |
| 8-14 days | 0.51 | 0.72 | 0.61 |
| 15-30 days | 0.43 | 0.67 | 0.56 |
| ≥30 days | 0.45 | 0.71 | 0.56 |
| Medical payment |  |  |  |
| UEBMI | 0.56 | 0.71 | 0.57 |
| NCMC/URBMI | 0.57 | 0.74 | 0.59 |
| Self-pay service | 0.57 | 0.65 | 0.89 |
| Others | 0.51 | 0.78 | 0.52 |
| Patterns of admission |  |  |  |
| Emergency | 0.55 | 0.74 | 0.64 |
| Outpatient | 0.57 | 0.74 | 0.65 |

Table S4. Annual per-person costs of common diseases (CNY)

| Cost category | All inpatients | The bottom 10% | The bottom 10%-50% | The top  10%-50% | The top  10% | Total cost proportion (%) |
| --- | --- | --- | --- | --- | --- | --- |
| All CVDs | 41281.82 | 4172.32 | 10330.30 | 52558.16 | 157099.10 | 100.00 |
| Common diseases |  |  |  |  |  |  |
| Hypertensive disease | 14692.67 | 4532.84 | 9910.32 | 39023.67 | 165037.90 | 1.96 |
| Coronary artery disease (CAD) | 33139.49 | 4493.97 | 9228.03 | 51993.31 | 139923.10 | 21.04 |
| Cardiomyopathy | 44121.59 | 4203.39 | 10950.73 | 48252.78 | 190518.50 | 1.13 |
| Heart failure | 30065.85 | 4354.94 | 11280.34 | 41156.16 | 163606.90 | 1.43 |
| Stroke | 37330.03 | 3860.39 | 12486.91 | 38113.02 | 175790.90 | 10.4 |
| Congenital heart disease (CHD) | 41675.50 | 3593.97 | 9620.35 | 47764.33 | 134108.10 | 2.82 |
| Myocardial infarction (MI) | 56117.76 | 4115.69 | 11841.38 | 54194.78 | 137103.00 | 4.66 |

Table S5. Composition of medical health care cost categories of inpatients with CVDs

| Cost category | All inpatients | The bottom 10% | The bottom 10%-50% | The top 10%-50% | The top 10% |
| --- | --- | --- | --- | --- | --- |
| Total health care cost, mean | 41281.82 CNY | 4172.32 CNY | 10330.30 CNY | 52558.16 CNY | 157099.10 CNY |
| Out-of-pocked cost (%), median (IQR) | 18.48 (5.11, 41.28) | 6.12 (1.95, 66.62) | 8.75 (4.38, 33.40) | 22.94 (9.61, 41.82) | 27.34 (14.62, 100.00) |
| Medical insurance cost (%), median (IQR) | 80.67 (56.83, 94.04) | 93.76 (29.79, 97.95) | 91.10 (65.20, 94.93) | 76.65 (55.23, 87.80) | 72.28 (0.00, 83.34) |
| Cost subcategory |  |  |  |  |  |
| Comprehensive medical service (%), median (IQR) | 2.15 (0.84, 4.44) | 4.53 (2.59, 7.16) | 3.31 (2.04, 5.42) | 0.84 (0.44, 2.22) | 1.23 (0.50, 2.34) |
| Diagnosis (%), median (IQR) | 23.43 (9.57, 55.89) | 62.81 (42.70, 74.27) | 53.87 (37.24, 63.32) | 11.21 (7.64, 18.17) | 7.31 (4.48, 10.69) |
| Treatment (%), median (IQR) | 2.19 (0.00, 5.74) | 0.00 (0.00, 0.00) | 0.00 (0.00, 0.63) | 4.37 (2.75, 6.45) | 4.80 (2.57, 11.30) |
| Drug (%), median (IQR) | 12.25 (4.93, 23.01) | 7.68 (14.03, 24.27) | 16.67 (11.03, 27.32) | 5.35 (2.81, 15.39) | 11.98 (3.64, 22.69) |
| Medical consumables (%), median (IQR) | 20.50 (2.98, 70.90) | 0.33 (0.00, 3.06) | 10.97 (1.67, 18.90) | 71.66 (38.77, 81.60) | 62.93 (44.66, 85.46) |

Table S6. Drivers for health care costs concentration

| Characteristics | Gini | ln (quantile (90 10)) |
| --- | --- | --- |
| Demography characteristics |  |  |
| Female | 0.030^***^ (0.023) | 0.256^***^ (0.011) |
| Age | 0.001 (0.001) | 0.021 (0.003) |
| Residence: Shanghai | 0.020^*^ (0.021) | 0.328^***^ (0.064) |
| Common disease |  |  |
| Hypertensive disease | -0.079^***^ (0.032) | -0.121^**^ (0.034) |
| Coronary artery disease (CAD) | -0.029^**^ (0.033) | -0.345^**^ (0.042) |
| Cardiomyopathy | 0.118^***^ (0.107) | 0.208^**^ (0.041) |
| Heart failure | 0.010^*^ (0.009) | 0.021 (0.022) |
| Stroke | 0.009^*^ (0.011) | 0.164^*^ (0.029) |
| Congenital heart disease (CHD) | -0.039^***^ (0.027) | -0.746^***^ (0.056) |
| Myocardial infarction (MI) | -0.247^***^ (0.324) | -1.232^***^ (0.091) |
| Disease characteristics |  |  |
| Comorbidity: cardiogenic disease | -0.010^*^ (0.009) | -0.054^**^ (0.001) |
| Comorbidity: non-cardiac disease | 0.001 (0.000) | 0.020 (0.046) |
| No. of comorbidities | 0.009^*^ (0.012) | 0.125^***^ (0.072) |
| Treatment |  |  |
| No. of operations | 0.031^***^ (0.036) | 0.082^***^ (0.023) |
| Level of operation | 0.061^***^ (0.079) | 0.021^***^ (0.020) |
| Medical service utilization |  |  |
| Length of hospital stay | 0.001 (0.007) | 0.076^**^ (0.044) |
| Medical payment: UEBMI/NRCMS/URBMI | 0.009 (0.008) | -0.023^**^ (0.002) |
| Medical payment: full out-of-pocket | -0.001 (0.002) | -0.002 (0.001) |
| Admission type: Outpatient | 0.001 (0.009) | 0.003 (0.031) |
| Average RIF | 0.560 | 1.007 |
| Adjusted $R^{2}$ | 0.121 | 0.216 |

Note: Bootstrap standard errors in parentheses. * p<0.1, ** p<0.05, *** p<0.01.

For different concentration measures, all the models produced consistent results with different insights. A higher concentration of health care costs resulted from an increase in the proportion of females. Specially, a 10% increase in the proportion of females would increase the Gini coefficient by 0.54% (0.030/0.560*0.1), and the costs gap between the 90^th^ and 10^th^ would increase 2.56%. The direction and magnitude of different common diseases on the concentration of health care costs differed. If the proportion of inpatients with cardiomyopathy increased by 10%, from 1% to 11%, the predicted Gini coefficient would increase by 2.11%, and the costs gap would increase 2.08%. Heart failure and stroke had similar positive effects on concentration as cardiomyopathy, but to a lesser extent. Hypertension, CAD, CHD, and MI did not significantly increase the concentration. In terms of comorbidity, with each additional comorbidity, the Gini coefficient will increase 0.009 and the costs gap would increase 12.5%.

Table S7. Estimated effect of selected key factors on the medical expenditures of coronary artery disease using quantile regressions (*n*=7,239)

| **Characteristics** | **10^th^ Quantile** | **50^th^ Quantile** | **90^th^ Quantile** |
| --- | --- | --- | --- |
| **Demography characteristics** |  |  |  |
| Sex |  |  |  |
| Female vs. Male | -216.8 (-399.6, -34.0) | -496.1 (-787.9, -204.2) | -5878.8 (-7880.1, -3877.5) |
| Age | Not select | Not select | Not select |
| Marriage: ref.= Unmarried |  |  |  |
| Married | Not select | Not select | Not select |
| Widowed or divorced | Not select | Not select | Not select |
| Residence |  |  |  |
| Native vs. non-native | Not select | Not select | -5878.8 (-9936.1, -400.1) |
| Occupation: ref.= Retired |  |  |  |
| Employed | Not select | Not select | Not select |
| Unemployed | Not select | Not select | Not select |
| Cardiogenic comorbidity |  |  |  |
| Yes vs. No | 323.3 (92.2, 544.3) | 966.6 (635.6, 1297.7) | 2333.6 (538.0, 4129.3) |
| Non-cardiac comorbidity |  |  |  |
| Yes vs. No | Not select | 363.5 (3.7, 723.3) | Not select |
| No. of comorbidities: ref.=0 |  |  |  |
| 1 | Not select | Not select | Not select |
| 2 | Not select | Not select | Not select |
| 3 | Not select | Not select | Not select |
| ≥4 | Not select | Not select | Not select |
| No. of medical procedures: ref.=0 |  |  |  |
| 1 | 3742.3 (3357.6, 4127.0) | 3627.1 (3076.2, 4178.0) | 4079.1 (2140.8, 6017.4) |
| 2 | 6542.0 (6037.1, 7046.9) | 13769.0 (11456.3, 16081.7) | 36720.8 (33306.2, 40135.4) |
| 3 | 9136.6 (8179.5, 10093.7) | 20025.9 (17673.6, 22378.1) | 41776.4 (36954.5, 46598.2) |
| ≥4 | 21376.6 (16701.9, 26051.2) | 30990.0 (29062.4, 32917.6) | 53441.4 (48878.0, 58004.8) |
| Level of medical procedure: ref.=I |  |  |  |
| II | 618.1 (219.2, 1017.0) | -321.4 (-609.9, -33.0) | 3942.0 (481.1, 7402.9) |
| III | 395.9 (-2.6, 794.4) | 420.9 (76.2, 765.6) | 9215.6 (5449.6, 12981.7) |
| IV | 11389.6 (7625.1, 15154.1) | 24217.4 (23614.5, 24820.3) | 30374.9 (26119.5, 34630.4) |
| **Medical service utilization** |  |  |  |
| Length of hospital stay | 66278.4 (5226.4, 7330.5) | 27736.4 (26040.6, 29432.1) | 41798.3 (36017.1,47579.5) |
| Medical payment: ref.= UEBMI |  |  |  |
| NRCMS/URBMI | Not select | 751.0 (108.8, 1393.2) | 14676.7 (9427.8, 19925.7) |
| Full out-of-pocket | Not select | -1385.2 (-1865.2, -905.2) | -7122.4 (-13301.6, -943.1) |
| Others | Not select | 229.2 (-412.5, 870.8) | 1811.4 (-2178.9, 5801.8) |
| Admission type |  |  |  |
| Outpatient vs. Emergency | -311.8 (-561.6, -62.0) | Not select | Not select |
| Proportion of out-of-pocket | Not select | 116.4 (11.0, 21.9) | 119.0 (50.6, 187.4) |

Table S8. Estimated effect of selected key factors on the medical expenditures of stroke using quantile regressions (*n*=3,177)

| **Characteristics** | **10^th^ Quantile** | **50^th^ Quantile** | **90^th^ Quantile** |
| --- | --- | --- | --- |
| **Demography characteristics** |  |  |  |
| Sex |  |  |  |
| Female vs. Male | Not select | Not select | Not select |
| Age | 882.4 (28.7, 136.1) | Not select | Not select |
| Marriage: ref.= Unmarried |  |  |  |
| Married | Not select | Not select | Not select |
| Widowed or divorced | Not select | Not select | Not select |
| Residence |  |  |  |
| Native vs. non-native | Not select | Not select | Not select |
| Occupation: ref.= Retired |  |  |  |
| Employed | Not select | Not select | Not select |
| Unemployed | Not select | Not select | Not select |
| Cardiogenic comorbidity |  |  |  |
| Yes vs. No | Not select | Not select | Not select |
| Non-cardiac comorbidity |  |  |  |
| Yes vs. No | Not select | Not select | Not select |
| No. of comorbidities: ref.=0 |  |  |  |
| 1 | Not select | Not select | Not select |
| 2 | Not select | Not select | Not select |
| 3 | Not select | Not select | Not select |
| ≥4 | Not select | Not select | Not select |
| No. of medical procedures: ref.=0 |  |  |  |
| 1 | Not select | 2688.9 (-1362.0, 6739.8) | 1550.2 (-8771.2, 11871.5) |
| 2 | Not select | 14041.6 (9087.3, 18995.9) | 21881.5 (6497.6, 37265.5) |
| 3 | Not select | 21334.9 (3739.5,38930.3) | 55196.3 (29789.0, 80603.6) |
| ≥4 | Not select | 51420.5 (39765.6, 63075.4) | 107281.3 (77806.0, 136756.7) |
| Level of medical procedure: ref.=I |  |  |  |
| II | 4916.6 (1358.3, 8474.9) | 11257.3 (8055.7, 14458.9) | 19684.9 (-3612.4, 42982.1) |
| III | 9878.4 (8489.4, 11267.5) | 18493.6 (13682.7, 23304.5) | 21362.3 (9824.7, 32900.0) |
| IV | 52645.1 (46865.8, 58424.4) | 74478.9 (67308.0, 81649.8) | 94061.9 (75973.9, 112149.9) |
| **Medical service utilization** |  |  |  |
| Length of hospital stay | 99882.2 (8939.4, 10825.0) | 21306.6 (18496.8, 24116.1) | 43253.3 (35948.3, 50558.3) |
| Medical payment: ref.= UEBMI |  |  |  |
| NRCMS/URBMI | Not select | Not select | Not select |
| Full out-of-pocket | Not select | Not select | Not select |
| Others | Not select | Not select | Not select |
| Admission type |  |  |  |
| Outpatient vs. Emergency | -3313.4 (-4385.5, -2241.4) | Not select | Not select |
| Proportion of out-of-pocket | Not select | Not select | Not select |

Table S9. Estimated effect of the key factors on the medical expenditures of CVDs using GLM

| **Characteristics** | The bottom 10% | The middle 45%-55% | The top 10% |
| --- | --- | --- | --- |
| **Demography characteristics** |  |  |  |
| Sex |  |  |  |
| Female vs. Male | -70.9 (-239.2, 97.4) | 15.7 (-270.3, 301.6) | -5297.9 (-10765.7, 169.9) |
| Age | 9.1 (2.0, 16.1) | -20.0 (-32.0, -8.0) | 446.0 (205.2, 686.9) |
| Marriage: ref.= Unmarried |  |  |  |
| Married | -89.8 (-505.8, 326.2) | -38.4 (-719.5, 642.8) | -3765.1 (-19149.8, 11619.5) |
| Widowed or divorced | 263.3 (-318.3, 844.9) | 306.2 (-505.3, 1117.7) | 3406.1 (-13773.5, 20585.8) |
| Residence |  |  |  |
| Native vs. non-native | -169.3 (-424.3, 85.7) | 200.6 (-234.1, 635.3) | -2322.2 (-9870.3, 5225.9) |
| Occupation: ref.= Retired |  |  |  |
| Employed | 109.5 (-112.8, 331.8) | -455.8 (-772.2, -139.4) | -2795.3 (-8889.5, 3298.8) |
| Unemployed | -38.4 (-629.6, 552.8) | -453.2 (-1100.4,193.9) | -6373.5 (-19490.6, 114189.8) |
| Cardiogenic comorbidity |  |  |  |
| Yes vs. No | 713.7 (419.4, 1008.0) | 664.4 (208.9, 1120.0) | -20601.1 (-155392.0,114189.8) |
| Non-cardiac comorbidity |  |  |  |
| Yes vs. No | 903.7 (592.7, 1214.6) | 398.0 (-65.4, 861.4) | -10602.5 (-145465.2, 124260.1) |
| No. of comorbidities: ref.=0 |  |  |  |
| 1 | -360.5 (-639.3, -81.8) | -321.7 (760.3, 116.8) | 14560.3 (-120613.7, 149734.4) |
| 2 | -93.3 (-366.3, 179.7) | 105.8 (-544.5, 333.0) | 7561.0 (-127612.6, 142734.5) |
| ≥3 | -104.3 (-386.1, 177.4) | -11.8 (-487.3, 463.6) | 5334.9 (-129862.5, 140532.2) |
| ≥4 | - | - | 11287.2 (-123691.9, 146266.3) |
| No. of medical procedures: ref.=0 |  |  |  |
| 1 | 247.8 (-29.4, 525.0) | 284.9 (-332.8, 902.6) | -65359.3 (-203281.4, 72562.8) |
| 2 | 706.7 (275.1, 1138.2) | 1210.5 (529.7, 1891.3) | -78399.6 (-216318.6, 59519.4) |
| 3 | 683.9 (-180.8, 1548.6) | 981.8 (247.0,1716.6) | -67197.7 (-216792.9, 59164.2) |
| ≥4 | 675.7 (-1049.4, 2400.9) | 1588.9 (716.9, 2460.9) | -67197.7 (-205112.4, 70716.9) |
| Level of medical procedure: ref.=I |  |  |  |
| II | 287.1 (-28.1, 602.2) | 150.6 (-288.6, 589.8) | 23507.8 (-6858.1, 53873.6) |
| III | 753.6 (431.3, 1075.8) | -143.9 (-556.9, 269.0) | 32635.6 (3333.9, 61937.3) |
| IV | 117.2 (-282.6, 517.0) | 923.2 (341.6, 1504.9) | 47024.6 (18091.5, 75957.6) |
| **Medical service utilization** |  |  |  |
| Length of hospital stay | 203.1 (-230.4, 636.5) | 380.4 (141.4,619.4) | 22542.5 (19565.6, 25519.3) |
| Medical payment: ref.= UEBMI |  |  |  |
| NRCMS/URBMI | 163.3 (-183.6, 510.2) | -322.4 (-856.3, 211.5) | -3291.9 (-12475.0, 5891.2) |
| Full out-of-pocket | 242.8 (-82.3, 567.9) | -183.5 (-695.4, 328.4) | 654.4 (-9903.8, 11212.6) |
| Others | -56.5 (-397.8, 284.7) | 1059.0 (468.5, 1649.5) | -12270.6 (-21943.4, -2598.0) |
| Admission type |  |  |  |
| Outpatient vs. Emergency | 203.8 (-128.7, 536.3) | -304.5 (-636.2, 27.2) | -10153.3 (-15895.8, -4410.9) |
| Proportion of out-of-pocket | -5.0 (-8.3, -1.7) | 3.6 (-1.5, 8.6) | -63.5 (-176.2, 49.3) |
